# Catheter-related bloodstream infection in end-stage kidney disease: a Canadian narrative review

**DOI:** 10.1186/s40697-016-0115-8

**Published:** 2016-05-05

**Authors:** Chris Lata, Louis Girard, Michael Parkins, Matthew T. James

**Affiliations:** Division of Infectious Diseases, Department of Medicine, University of Calgary, Calgary, Canada; Division of Nephrology, Department of Medicine, University of Calgary, Calgary, AB T2N 2T9 Canada; Division of Nephrology, Department of Community Health Sciences, University of Calgary, Calgary, AB T2N 2T9 Canada

**Keywords:** Haemodialysis, Bacterial infection, Bacteremia, Complications, Prevention, Treatment, Risk factors

## Abstract

**Purpose of the review:**

Patients with end-stage renal disease (ESRD) are at a high risk of bacterial infection. We reviewed publications on risk factors, prevention, and treatment paradigms, as well as outcomes associated with bacterial infection in end-stage kidney disease. We focused in particular on studies conducted in Canada where rates of haemodialysis catheter use are high.

**Sources of information:**

We included original research articles in English text identified from MEDLINE using search terms ‘chronic kidney failure’, ‘renal dialysis’, or ‘chronic renal insufficiency’, and ‘bacterial infection’. We focused on articles with Canadian study populations and included comparisons to international standards and outcomes where possible.

**Findings:**

Bacterial infections in this setting are most commonly due to Gram-positive skin flora, particularly *Staphylococcus*, with methicillin-resistant *Staphylococcus aureus* (MRSA) carrying a poorer prognosis. Interventions that may decrease mortality from sepsis include a collaborative care model that includes a nephrology team, an infectious disease specialist, and use of standardized care bundles that adhere to proven quality-of-care indicators. Decreased infectious mortality may be achieved by ensuring appropriate antibiotic selection and dosing as well as avoiding catheter salvage attempts. Reduction in bloodstream infection (BSI) incidence has been observed with the use of tPA catheter-locking solutions and the use of mupirocin or polysporin as a topical agent at the catheter exit site, as well as implementing standarized hygiene protocols during catheter use.

**Limitations:**

There has been a paucity of randomized controlled trials of prevention and treatment strategies for catheter-related BSIs in haemodialysis. Some past trials have been limited by lack of blinding and short duration of follow-up. Microbiological epidemiology, although well characterized, may vary by region and treatment centre.

**Implications:**

With the high prevalence of catheter use in Canadian haemodialysis units, further studies on long-term treatment and preventative strategies for BSI are warranted.

## ABRÉGÉ

**But de la revue:**

Les patients souffrant d’insuffisance rénale terminale (IRT) sont à risque élevé de contracter une infection bactérienne. Nous avons effectué une revue des publications faisant état des facteurs de risque, des paradigmes de prévention et de traitement, ainsi que des pronostics associés à la contraction d’une septicémie en situation d’IRT. On a porté une attention particulière aux études conduites au Canada, où le taux d’hémodialyse par accès vasculaire est élevé.

**Sources:**

Nous avons inclus tous les articles rédigés en anglais répertoriés sur MEDLINE qui répondaient aux critères de recherche suivants : « hémodialyse », « insuffisance rénale chronique » et « infection bactérienne ». L’accent a été mis sur les articles portant sur des études s’étant tenues au Canada, en incluant des comparaisons aux pronostics et aux standards internationaux lorsque possible.

**Constatations:**

La plupart des infections bactériennes dans ce contexte particulier sont attribuables à des bactéries Gram positif, plus spécifiquement à *Staphylococcus aureus* résistant à la méthicilline (SARM), l’agent infectieux présentant le moins bon pronostic. Un modèle de soins collaboratifs, basé sur un plan standardisé et conforme à des indicateurs de la santé éprouvés, où interviennent l’équipe de néphrologie et un spécialiste des maladies infectieuses, figure parmi les interventions pouvant réduire le risque de mortalité par septicémie. On a également observé qu’on pouvait réduire le taux de mortalité associé à ce type d’infections en prescrivant l’antibiotique adéquat à la dose optimale, de même qu’en évitant la réutilisation du cathéter. De plus, on a constaté que l’emploi d’une solution de blocage contenant du t-PA (activateur tissulaire du plasminogène) dans le dispositif de verrouillage du cathéter ainsi que l’application topique de mupirocine ou de polysporin au point d’émergence du cathéter permettaient de réduire l’incidence des infections.

**Limites de l’étude:**

Il existe très peu d’essais cliniques randomisés rapportant des stratégies de prévention ou de traitement des bactériémies liées à l’utilisation de cathéters pour l’hémodialyse.

**Conséquences:**

Étant donné la prévalence élevée d’utilisation de cathéters dans les unités de dialyse au Canada, il est suggéré d’effectuer des études supplémentaires afin d’élaborer des stratégies à long terme pour la prévention et le traitement des infections du sang.

## What was known before

Bacterial infection represents a significant cause of morbidity and mortality in patients with end-stage renal disease (ESRD). The incidence of infection is highest in those patients who use a catheter for haemodialysis vascular access, which is common in Canada.

## What this adds

We undertook a collaborative review of the literature, involving specialists in both nephrology and infectious diseases, to characterize the epidemiology of bloodstream infections in ESRD, and literature on best practices for prevention and treatments to improve outcomes.

## Background

Bacterial infections are a common cause of morbidity and mortality in patients with kidney disease. Much of the literature about bloodstream infection in the setting of kidney disease has focused on infection related to dialysis access. In this article, we review the incidence, risks, management, and outcomes of catheter-related bloodstream infection (CRBSI) in patients with ESRD. We focus on the literature addressing this problem in Canada.

## Review

### Incidence and risk factors

CRBSI is one of the most common forms of bacterial infection in patients receiving haemodialysis (HD), with an estimated incidence of 1.2–2.5 per 1000 patient-days [1–5]. Bacteremia in patients with ESRD may be under-ascertained in many general studies based on methodological criteria and infection classification. This is illustrated by a large multicentre study [6] comparing incidence and risk factors for healthcare-associated bacteremia and community-acquired bacteremia. Here, the study excluded patients with blood cultures growing coagulase-negative *Staphylococcus* (CONS) species as presumed contaminants. However, a significant proportion of CONS isolates likely represent true bacteremia rather than contamination in patients with ESRD and HD catheters. Even within the HD population, there is significant variance in how CRBSI is defined and reported in the literature [7]. Nonetheless, published bacteremia rates are consistently much higher in patients with ESRD relative to the general population (Table 1) [2]. In Canada, the incidence of community-associated bacteremia varies by city, but population studies estimate rates from 0.22 to 0.28 per 1000 patient-days, which is 5–10 times lower than that of patients receiving HD [8].

**Table 1:** Incidence of CRBSI from select cohort studies of patients on HD

Country	Incidence^a^	Study information	Reference
Canada	1.2/1000 Pt-days	*N* = 527, half of the patients were new HD starts, the other half were continuing HD with access change.	[1]
USA	2.5/1000 Pt-days	*N* = 47, inpatients admitted to hospital	[2]
USA	0.4/1000 Pt-days	*N* = 445, outpatients, *S. aureus* bacteremia only	[3]
Spain	1.6/1000 Pt-days	*N* = 51, outpatients, monitored by surveillance cultures^b^	[4]
Canada	1.6/1000 Pt-days	*N* = 94, outpatients, tunnelled cuffed catheters, surveillance cultures	[5]

^a^Incidence values were converted from studies to patient-days

^b^Surveillance cultures were blood cultures taken from catheter lumen or exit site at regular intervals and were repeated along with peripheral blood cultures when CRBSI was suspected

Most literature relating to bacteremia in the ESRD population focuses on patients that require HD. However, the risk of BSI is also elevated in those with chronic kidney disease (CKD) who do not require dialysis. A large multicentre Canadian cohort study of patients older than 66 years of age showed that rates of bacteremia increased with decreasing estimated glomerular filtration rate (eGFR) even in the absence of dialysis [9]. This suggests that CKD is an independent risk factor for infection in addition to risks conferred from that of vascular access.

Patients with ESRD experience repetitive exposure to hospital and healthcare environments, which is an independent risk factor for nosocomial infections. Patients receiving chronic HD have been shown to have a higher incidence of nosocomial infection compared to hospitalized patients not requiring dialysis in the same study period at a single centre (9.1 vs. 3.8/1000 patient-days, RR 2.4, *p* < 0.001) [2]. In a multicentre study by Kollef et al. examining incident BSI in the general population, it was found that those admitted with healthcare-associated bacteremia were more likely to have higher severity of disease presentation determined by acute physiology score, higher risk of mortality (HR 2.80, 95 %CI 1.5–5.1, *p* < 0.001), and significantly longer median duration of hospital stay (8 vs. 7 days, *p* = 0.03) [6]. Chronic hospital exposure also tended to change the bacterial aetiology of infection, where patients with healthcare-associated bacteremia had a higher tendency toward methicillin-resistant *Staphylococcus aureus* (MRSA), *Enterococcus* spp., *Pseudomonas aeruginosa*, and *Klebsiella pneumoniae* infections than those with community-acquired infections. Interestingly, these types of infections are also common in patients with ESRD, suggesting that exposure to hospital environments likely plays an important role in the types of infections seen in this population.

Patients with ESRD requiring chronic dialysis are at a higher risk both for developing true infections and becoming colonized with bacterial strains that gain drug resistance over time [10]. These risks are likely conferred by decreased innate immunity [11, 12], chronic hospital exposures, and dialysis access itself (the most common source for bacteremia) in the context of frequent antibiotic exposures. This represents a significant source of morbidity, potential mortality, and cost in the care of patients on HD [13–15].

### Relationship to HD vascular access

For patients receiving HD, the type of access, and the manner in which it is utilized and maintained influences BSI risk (Table 2).

**Table 2:** Risk factors for bacteremia relating to access type and patient status

Country	Risk factor	Relative risk bacteremia	Reference
Canada	Prior access infection (vs. no prior access infection)	3.33 (95 %CI 2.1–5.2)	[1]
Canada	30 days post-access type change (vs. continued HD modality)	1.56 (95 %CI 1.02–2.4)	[1]
Canada	Cuffed catheter (vs. AVF)	8.49 (95 %CI 3.0–23.8)	[1]
Canada	Uncuffed catheter (vs. AVF)	9.87 (95 %CI 3.5–28.2)	[1]
	Catheter vs. AV Graft	7.6 (95 %CI 3.7–15.6)	[19]
USA	Chronic HD vs. non-HD patients	1.8 (95 %CI 1.1–3.1)	[2]
France	Immunosuppressive therapy (vs. no immunosuppressive treatment)	3.0 (95 %CI 1.0–6.1)	[19]
France	Prior access infection (vs. no prior access infection)	7.3 (95 %CI 3.2–16.4)	[19]
Denmark	*S. aureus* bacteremia with CVC (vs. none); general inpatient population not specific to ESRD	6.9 (95 %CI 2.8–17.0)	[20]

1. Access type

It has long been recognized that the use of catheters and arteriovenous grafts for HD access is associated with a higher risk of BSI than arteriovenous fistulas (AVFs) [16]. However, with an increasing prevalence of ESRD and advancing age of the Canadian dialysis population, catheter use is becoming more common in Canada [17, 18]. In Canada, the prevalence of AVFs in the HD population dropped from 54 % in 2002–2003 to 50 % in 2005–2007, despite increased infection risk associated with catheters and grafts (Table 2) [19, 20]. Furthermore, the international Dialysis Outcomes and Practice Patterns Study (DOPPS) has observed similar trends in access type in several countries. As of 2011, data from phase 4 of DOPPS showed that Canada has the highest prevalence of patients dialysed via permanent catheter of all countries studied at 49.1 % and correspondingly also has the lowest AVF prevalence at 45 % [18, 21]. Some literature has suggested that elderly patients may be at lower risk of CRBSI than younger patients. In a study comparing patients ≥75 years old to those 18–74, elderly patients had a 67 % lower adjusted risk of CRBSI, hazard ratio of 0.33 (95 %CI 0.20–0.55) [22]. The authors hypothesized decreased mobility and apocrine gland function as possible aetiologies for the lower incidence of infection in elderly patients.

There are several patient characteristics that make it more challenging to achieve a functioning AVF. They include female gender, advanced age, diabetes, and peripheral vascular disease, which are all common in the HD population. Additionally, a Canadian study found that two thirds of patients requiring HD refused creation of an AVF. The most common reasons cited were concerns regarding pain, bleeding, aesthetics, a negative experience with a previous AVF attempt (personally or with another patient), and misconception about the vascular access team’s comfort with AVF management [23]. Another study conducted by the DOPPS found that Canada has the highest patient preference for catheter use for HD access [24]. While a focus on achieving higher rates of successful AVF remains an important goal of HD vascular access programmes in Canada, it is important to note that the heightened risk of CRBSIs associated with the use of catheters cannot be eliminated entirely.

2. Catheter-related infections and management strategies

Although international guidelines recommend that the majority of HD accesses should be AVF, there are many factors that prevent achievement of a functioning AVF. As catheters are known to be associated with a high risk of infection, a large body of research has focused on strategies to prevent and treat catheter-related infections in HD. Risk factors for infection and poor prognostic factors in patients with catheter-related infections are summarized in Table 3 [25–27].

**Table 3:** Bacteremia risk factors and significant prognostic factors for poor outcomes in patients with ESRD dialysing with tunnelled cuffed catheters

BSI risk factors	Mortality risk factors
^a^Recent modality change [1]	*Staphylococcus aureus* colonization
Previous bloodstream infection [1]	Failed salvage [26]
Diabetes mellitus [27]	Hypoalbuminemia [20]
^a^New access [1]	Abnormal/infected exit site [25]
Poor hygiene (subjective assessment) [78]	

^a^Infectious risk during a 6-month follow-up of new vascular access or catheter exchange

Management of CRBSI often includes empiric broad-spectrum antibiotics along with any of the following: (1) advancing to use of an arteriovenous access (AVF/AVG) if it has been created and is mature for use, (2) catheter withdrawal with delayed replacement, (3) catheter withdrawal and immediate replacement, or (4) catheter salvage (current line remains in place) with a course of IV antibiotics that varies in duration and is guided by the type of organism and antimicrobial sensitivities. A large prospective observational study, which included middle-aged patients in whom diabetes and hypertensive nephropathy comprised the majority of ESRD aetiology, compared outcomes with these approaches (Table 4). The primary endpoint was treatment failure defined as a composite of re-infection with the same organism within 6 months or death by sepsis. Attempted salvage of the current catheter was associated with the highest risk of treatment failure [25]. Additional research by Ashby et al. [26] suggested that salvage therapy is a viable strategy in those presenting with non-severe sepsis and with good 48-h response to empiric antibiotics indicated by culture negativity. In this study, two thirds of the patients did not require catheter replacement. However, compared to a strategy of catheter withdrawal, the salvage approach was associated with a significantly higher risk of treatment failure at 6 months (33 vs. 8 %, *p* < 0.001). Additionally, repeated attempts at salvage therapy in patients with recurrent infections were even less likely to be successful. Thus, catheter removal, which attains source control, appears to be the most efficacious and safest approach to treatment. However, availability of alternate vascular access sites and co-morbid conditions may make standardized recommendations for catheter management challenging. Thus, algorithmic approaches to CRBSI should consider the nature and severity of the infection, comorbidity, and vascular access history of the patient. The role of standardized criteria to guide catheter salvage attempts warrants further study.

**Table 4:** Treatment failure from bacteremia recurrence and complications with CRBSI with different management strategies [25, 26] (adapted)

Treatment Strategy	Treatment Failure (%)	Infectious Complications^*d*^ (%)	Septic Death (%)
Salvage (6-wks IV antibiotics)^*a*^	26–33	2–13	6
Immediate Catheter Replacement^*b*^(2-wks IV antibiotics)	3	3	0
Removal with Delayed Replacement^*c*^	11	5	4
Changed Access Type	5	9	2
ANOVA significance (*P*-value)	0.002	0.33	0.45

^*a*^Salvage was only attempted in CRBSI where clinical presentation was not severe (defined as no features of severe sepsis) and where adequate treatment response was observed within 48 hours of antibiotic initiation (defined as being afebrile with resolution of symptoms). ^*b*^Removal and over-wire exchange or new site within 24-48 hours of development of severe features of infection; re-implantation was done without waiting for blood culture negativity. ^*c*^Delayed re-implantation of catheter for a minimum 1-week interval after culture negativity observed. ^*d*^Infectious complications included septic pulmonary emboli, abscess, and osteomyelitis

**Fig. 1:**
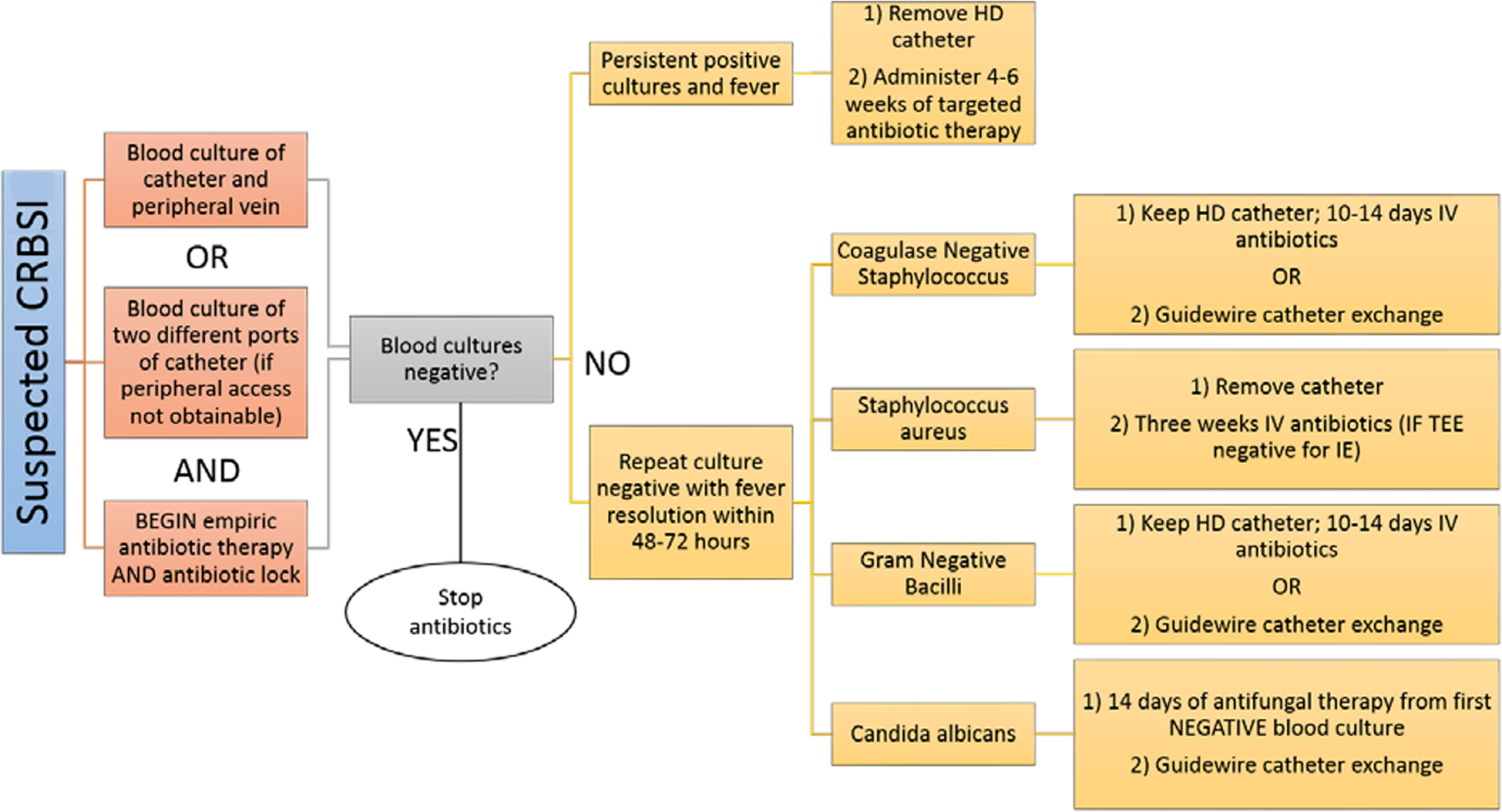
Guidelines for treatment of suspected CRBSI in patients using a permanent catheter; adapted (*Persistent positive cultures should prompt search for metastatic foci for source control, and recommended duration begins when source control is obtained; *Day 1 of antibiotics is from the first day of blood culture negativity) [28]

General guidelines put forth by the Infectious Diseases Society of America for CRBSI were last updated in 2009 [28] and may be utilized in circumstances where HD catheter infections are encountered. It should be noted that catheter salvage is not recommended in cases of severe sepsis, endocarditis (discussed separately), hemodynamic instability, or in the case of bacteremia persisting >72 h on appropriate therapy. Infections with the specific pathogens *S. aureus*, *P. aeruginosa*, fungi, or mycobacteria are also indications to remove the catheter and not attempt salvage. Special considerations for patients requiring HD in these circumstances are outlined in Fig. 1; however, evidence supporting these recommendations in the HD population are limited. In a study by Ashby et al. [26], four patients who underwent delayed re-implantation with use of a temporary dialysis died due to sepsis, whereas no such deaths were observed with immediate replacement. However, the number of subjects in the immediate withdrawal and replacement group was too small to draw definite conclusions. Here, delayed replacement meant a minimum of 1 week from blood culture negativity to replacement of a dialysis catheter, whereas immediate replacement occurred if resolution of symptoms did not occur after 48 h or if features of severe sepsis were observed. Another study comparing these management strategies did not find significant differences in re-infection or septic mortality [29]. It is important to note that this study did not include patients with risk factors for poor outcomes including exit site infection or severe sepsis. Those individuals that developed severe sepsis were treated with antibiotics and prompt catheter removal, creating a selection bias and limiting the generalization of findings to patients with CRBSI and features of severe sepsis.

Decisions to attempt salvage of catheters infected with Gram-positive organisms other than *S. aureus* largely depend on the clinical status of the patient and the availability of alternate vascular access options. For example, there is evidence that this technique may be employed when bacteremia is due to CONS, although there is a 6.6-fold increased risk of recurrence compared with catheter exchange [30]. The success of catheter salvage may be improved by concomitant use of antibiotic locking solutions with intravenous treatment and are recommended by the Infectious Diseases Society of America (IDSA) if catheter salvage is to be attempted [28]. However, evidence surrounding this B-level recommendation is pre-dominantly drawn from studies of patients with catheters for total parental nutrition, and there is a paucity of comparative studies in patients receiving HD [31, 32]. Thus, catheter salvage with antibiotic locking solutions warrant further dedicated study in HD.

The best clinical outcomes in the management of CRBSI are achieved through adherence to clinical guidelines and early collaborative involvement of infectious disease specialists in the care team. This was illustrated by a prospective multicentre trial where six quality-of-care indicators for *S. aureus* bacteremia were defined through a systematic review of the literature (see Table 5) [33]. These factors were monitored in a pre-intervention period for adherence as well as infection outcomes and compared to an intervention period where infectious disease specialists were consulted automatically for hospitalized patients at the onset of a positive *S. aureus* blood culture. Both adherence to quality-of-care indicators and 30-day mortality improved significantly with the intervention (OR 0.56, 95 %CI 0.34–0.93), and although the study was not specific to CRBSI, patients with catheters represented 39 % of the observed cohort. A 2-year multi-centre randomized prospective study specific to patients receiving HD via a tunnelled catheter in an outpatient setting showed significant improvement in outcomes utilizing a collaborative care model involving an infection control manager, who was a nurse trained in current catheter management guidelines. Here, infection recurrence (OR 0.28, 95 %CI 0.09–0.8, *p* = 0.015) and sepsis-related death (0 vs. 6 %) were reduced, and there was a 45 % reduction in attempted catheter salvage also observed in the centres’ treatment practice [34]. Thus, the utilization of a collaborative model that involves automatic infectious disease consultation utilizing bundled guideline-based care that is early and automatic may significantly improve infection outcomes.

**Table 5:** Clinical quality-of-care indicators in patients presenting with *S. aureus* bacteremia (adapted from Cortes et al., *CID* 2013; 57, 1225–1233 [33])

Quality-of-care Indicator	Definition
Follow-up blood cultures	Repeat blood cultures performed 48 hours after antibiotic initiation *regardless* of clinical evolution
Early source control	Removal of non-permanent catheter if suspected or confirmed source within 72 hours; exclusion of metastatic foci of infection
Echocardiography in patients with clinical indications	Performance of echocardiography in complicated bacteremia or patients with predisposing conditions for endocarditis
Early use of intravenous cloxacillin for MSSA bacteremia	Definitive treatment with cloxacillin (2 g IV q6h) within 24 hours of culture sensitivities. In patients on HD, cefazolin 2 g after each dialysis session was acceptable
Adjustment of vancomycin dose according to trough levels	Trough levels obtained in all patients treated at least 3 days, with adjustment of dose to target trough level of 15-20 mg/L
Treatment duration according to complexity of infection	Duration at least 14 days in uncomplicated bacteremia and 28 days in complicated cases

3. CRBSI epidemiology and empiric therapy

Information from several studies has shed light on the common bacterial species that cause CRBSI in patients receiving HD, which is important to inform empiric antibiotic selection. There is substantial variation in the distribution of causative agents of infections according to geographical area and dialysis site, which has been illustrated in both American [14] and Canadian studies [35]. However, the general trend across sites is for Gram-positive cocci, particularly CONS and *S. aureus*, to cause the majority of infections. It is recommended that local bacterial resistance patterns guide empiric antibiotic choice, particularly with respect to MRSA, where vancomycin may be used empirically unless a high prevalence of isolates with a vancomycin minimal inhibitory concentrations (MICs) >2 μg/mL exists, in which case alternate agents such as daptomycin or linezolid should be used first line [28].

Notably, Canadian epidemiological data suggest a low incidence of infection due to Gram-negative species relative to American studies (8–10 vs. 5–45 %) [1, 14]. Both a prospective multi-centre national Canadian study and a Quebec province-wide surveillance programme (SPIN-HD) show that the vast majority of isolates in CRBSI for patients with tunnelled cuffed catheters were Gram-positive (Table 6) [35, 36]. However, there was relatively wide variation in the distribution of causative pathogens depending on the dialysis site, which may have been attributable to both a variation in prevalence of access type and differences in access maintenance policies.

**Table 6:** Incidence of bacteremia in haemodialysis patients using permanent catheters by pathogenic species in a national prospective Canadian study and Quebec surveillance programme

Organism	Incidence^a^ (%)	SPIN-HD Incidence^b^ (%)
*Staphylococcus aureus*	31.9	55
*Coagulase negative staphylococci*	40.4	14
*Enterococcus spp.*	7.5	5
*Streptococcus spp.*	2.1	
*Enterobacter spp.*	3.2	
*Pseudomonas spp.*	2.1	3
*Candida spp.*	3.2	1
*Klebsiella spp.*	1.1	4
*Corynebacterium spp.*	2.1	
*Escherichia coli*	1.1	1
*Stenotrophomonas maltophila*	2.1	
*Other spp.*	3.2	16^c^
Incidence rate (per 1000 patient-procedures)	3.1	3.7

^a^Adapted from Taylor et al., 2002

^b^Adapted from 2014 to 2015 SPIN-HD surveillance data [36]

^c^Includes grouped enteric and anaerobic organisms

Empiric antibiotics for treatment of suspected bloodstream infection in patients receiving HD are typically selected to cover Gram-positive, Gram-negative, and anaerobic species but are primarily focused on coverage of *S. aureus* (and in particular MRSA) because of its association with poor outcomes. The specific antimicrobial drugs utilized should be tailored to the known antimicrobial resistance patterns of the region and patient colonization status (i.e. vancomycin-resistant *Enterococcus* (VRE) and MRSA). Often, therapy is governed by local dialysis centre policies and treatment algorithms, developed based on local incidence and prevalent pathogens and resistance patterns of the specific site in question.

Many empiric regimens include vancomycin for Gram-positive coverage due to high rates of MRSA infection in the HD population. However, this strategy may have limitations. In a small prospective study which utilized vancomycin heavily as empiric therapy, there was a 44 % complication rate of bloodstream infection, which included osteomyelitis, infective endocarditis, and death, within a 3-month period of initial infection [3]. In cases where blood cultures reveal methicillin-sensitive *S. aureus* (MSSA), there is increasing evidence that continued use of vancomycin predisposes patients to a higher risk of treatment failure than with other bactericidal anti-Staphylococcal antibiotics. A prospective multicentre study identifying dialysis patients with MSSA bacteremia revealed that, although those treated empirically with vancomycin tended to be younger and have less metastatic complication compared to those treated with cefazolin, there was significantly a higher risk of treatment failure (31.2 vs. 13 %, *p* = 0.02) [37]. Additionally, a large retrospective analysis of antibiotic use in *S. aureus* bacteremia revealed that those with MSSA were often kept on treatment with vancomycin rather than switched to cefazolin, despite culture results. In this study, those treated with cefazolin were significantly less likely to require hospitalization or die from infection (HR 0.62, 95 %CI 0.46–0.84) or to develop sepsis (HR 0.52, 95 %CI 0.33–0.89) [38]. Another prospective study utilizing a collaborative care model with an infectious disease consultant also showed reduction in recurrent infection and septic death, where those in the collaborative care group were significantly less likely to be treated with an inappropriate antibiotic or dose (13 vs. 37 %, *p* < 0.001) [34].

Why vancomycin is inferior to other parenteral anti-Staphylococcal agents such as cefazolin and cloxacillin is likely multifactorial. Vancomycin, like the beta-lactam antibiotics, is a cell wall inhibitor; however, the bactericidal activity of vancomycin is significantly slower. Vancomycin dosing in those patients with ESRD also presents a challenge in attaining an adequate drug level, and lack of initial bolus (recommended at 15–20 mg/kg) [39] dose can delay the time to attaining adequate drug levels in the serum. Furthermore, MIC creep has been observed in MRSA, such that some isolates are progressively less susceptible to the effects of vancomycin. Indeed, many groups have documented that heterogeneous vancomycin intermediate *S. aureus* (hVISA) exist in complicated infections (subpopulations of isolates having higher MICs against vancomycin). hVISA and even vancomycin resistant *S. aureus* (VRSA) have been reported in dialysis patients [40]. This highlights the need to use vancomycin judiciously and correctly in the ESRD population, where its empiric use is warranted but where culture sensitivity data requires timely follow-up to facilitate a switch to alternate antibiotics where sensitivity results indicate they would be more appropriate [40, 41].

These studies illustrate the importance of obtaining adequate and timely blood cultures to facilitate speciation of the aetiological agent. Subsequent follow-up of cultures and tailoring of therapy to the best suited antimicrobial agent appears to be important to improve infectious outcomes in patients on HD.

4. Infective endocarditis as a complication of bacteremia

One of the most serious complications of BSI in the dialysis population is infective endocarditis (IE). In an international prospective study, HD was identified as a significant independent risk factor for hospital-associated endocarditis, present in 30 % of cases [42]. With an incidence estimated at 267/100,000 person-years in the American HD population [43], IE occurs far more commonly in patients receiving HD than in the general population (estimated incidence 1.7–6.2/100 000 person-years) [44]. IE represents a significant source of morbidity and mortality in patients with ESRD, with in-hospital mortality estimated at 24 % [45], and 1-year mortality reported between 52 and 62 % [45–47]. A large population-based retrospective cohort showed that in-hospital and long-term survival rates have changed little since 1977 [45]. The higher incidence of IE in patients undergoing HD likely relates to the higher incidence of bacteremia relating to frequent vascular access [46], but other contributors such as vascular and cardiac valvular changes associated with long-term HD are also thought to be contributors [48]. Although antibiotics are the mainstay of treatment, surgery may be required in some cases, and one small case-control study described that early surgical valve repair was a predictor of survival in patients receiving HD (OR 5.39, 95 %CI 1.3–17.6, *p* = 0.023) [49].

The most common aetiological microorganism for IE in the HD population is *S. aureus*, and a relatively large proportion (24–42 %) of these infections are methicillin-resistant organisms [46, 49]. IE due to MRSA has been shown to be associated with high mortality in patients receiving HD (HR 2.43, 95 %CI 1.18–5.00, *p* = 0.016). Other risk factors that have been associated with mortality following endocarditis in the HD population include advancing age, diabetes as a cause of kidney disease, and congestive heart failure [45]. Given the high risk of endocarditis and poor outcomes, many algorithms for management of CRBSI suggest investigation with echocardiography in patients with *S. aureus* bacteremia and longer treatment courses of antibiotics.

5. CRBSI prevention and risk reduction

Since patients receiving HD are at a high risk of BSI and the consequences of these infections can be serious, there has been substantial interest in identifying strategies to prevent bloodstream infection. Much of the research has been focused on prevention of catheter colonization. Colonization certainly precedes bacteremia in CRBSIs, and so it seems logical to attempt surveillance of patients receiving dialysis via a catheter in an attempt to identify and prophylactically treat those who showed evidence of nascent bacterial growth. One small prospective study involving 56 patients with new catheters who were free of infection at study onset monitored patients every 15 days with both venous and arterial lumen blood cultures. The study showed increased likelihood of CONS infection with preceding surveillance blood culture positivity. The study controlled for and ruled out culture contamination by matching time to positivity and using *Staphylococcus epidermidis* bacterial strain typing to link colonization to later bacteremic presentations [4]. However, this was a small study and did not identify colonization leading to infection by more virulent strains, such as *S. aureus*. A larger Canadian trial, which utilized surveillance swab cultures of exit sites, examined the effect of topical antimicrobial treatment of those found to be swab-positive versus ongoing clinical surveillance [5]. Those in the treatment arm received a 2-week course of prophylactic topical antibiotics at the exit site. Interestingly, the authors found there were significantly higher rates of exit site infection as well as bacteremia in those who were treated with topical antimicrobials. The authors hypothesized increased catheter site manipulation and alteration of the natural skin flora at exit sites as possible mechanisms for the unexpected increase. Additionally, the monthly prevalence of positive exit site cultures was 15 %, making this strategy both costly and ineffective, likely by failure to eradicate colonizing bacteria within the catheter.

Preventing colonization of the exit site and catheter hub has been shown to significantly reduce CRBSI in a large multicentre trial, in which catheter care was standardized. This included use of chlorhexidine at the exit site prior to HD initiation and 70 % alcohol pad scrubbing prior to manipulation of the catheter hub. When compared to usual care, this ‘scrub the hubs’ technique led to a significant risk reduction in CRBSI (RR 0.79, 95 %CI 0.78–0.81). This standardized use of aseptic technique resulted in lasting reductions in CRBSI over 1 year of follow-up relative to the usual care and also significantly reduced the need for new intravenous antibiotic starts.

Topical agents to eradicate nasal carriage of *S. aureus* appear to be effective in short-term studies. Notably, a significant reduction in the incidence of *S. aureus* bacteremia was observed compared to the 2-year historical incidence (0.04 vs. 0.25 per patient-years) in a single centre that eradicated positive nasal carriage with mupirocin ointment [50]. A systematic review and meta-analysis has examined the effects of mupirocin for nasal eradication of MRSA as well as mupirocin application at the exit site for prevention of *S. aureus* infection specifically. In those undergoing HD, an 80 % (95 %CI 65–89 %) relative risk reduction was calculated for *S. aureus* infection. The majority of benefit was derived from the prevention of bacteremia, with a smaller component from prevention of exit site infections [51].

Similar research has been conducted to examine the role of nasal swabs and MRSA nasal eradication in outpatients receiving HD. In one small study, those with positive MRSA nasal carrier status had greater than four-fold risk of *S. aureus* infection and a five-fold risk of infection-related death compared to non-colonized patients [52]. Furthermore, persistent nasal colonization resistant to nasal eradication therapy has been associated with increased mortality [53]. A Canadian randomized trial compared polysporin™ (containing polymyxin B, bacitracin, and gramicidin) and mupirocin intranasal applications in the eradication of MRSA in a complex, high-morbidity inpatient population that included patients with renal disease [54]. The rates of eradication and re-colonization were compared between these agents when used in conjunction with 7 days of chlorhexidine body washing. Mupirocin therapy yielded significantly greater eradication (65 vs. 31 %, *p* = 0.001); however, at the end of follow-up at 12 weeks, eradication levels had dropped in both groups (30.8 vs. 2.8 %, *p* = 0.001). Furthermore, in both groups where surveillance swabs had become positive again, there was significant development of mupirocin resistance (10 %), which is a documented phenomenon due to selective pressure in centres utilizing these practices [55–57]. Unfortunately, the intervention duration was limited to 7 days, and extended therapy was not tested. Similarly, the use of prophylactic topical antimicrobial agents at HD catheter exit sites has also been evaluated in several trials. In a meta-analysis of these randomized trials, both polysporin™ (RR 0.25, 95 %CI 0.12–0.56) and mupirocin (0.19, 95 %CI 0.08–0.45) application to the catheter exit site reduced bacteremia [58]. Given the promising results of short-term trials of MRSA eradication and catheter exit site topical antibiotic use, further prospective study is required to answer questions of long-term efficacy and bacterial resistance when these approaches are used in an enduring fashion.

In part, the additional risk of infection in those with catheters lies in the formation of biofilms along internal catheter surfaces, which are resistant to antibiotic therapy and provide a continually evolving source for septic emboli and re-infection after an attempt at antibiotic therapy [26]. Catheters are also prone to clotting, which is a major cause of access failure, and thus, standard therapy includes ‘locking’ these lines with an anticoagulant solution in between dialysis sessions. Unfortunately, heparin does not have antimicrobial properties, and there is evidence that it may actually stimulate *S. aureus* biofilm proliferation [59]. Thus, much research has been devoted to reducing both line clotting and infection through different locking solutions and catheter lining materials [14, 60]. Although many solutions appear to reduce colonization and infection (see Table 7) [61–67], there is controversy regarding the use of antibiotic-based solutions for fear of developing antimicrobial resistance [68, 69] and drug side effects, such as gentamicin ototoxicity [70], with prolonged exposure.

**Table 7:** Trials of haemodialysis catheter-locking solutions or catheter materials for CRBSI prevention

Intervention^a^	Population size and characteristics	Significant infection reduction?	Limitations and attributes	Ref
Cloxacillin vs. heparin	100 (uncuffed temporary lines)	Yes (0.5 vs. 7.8/1000 catheter-days)	Small sample, short median catheter life (60 days)	[61]
Bismuth-coated catheters	77 (uncuffed catheters)	No (significantly reduced catheter colonization in CFU/mL, 63 vs. 3.5, *p* < 0.001)	Majority of catheters removed as HD no longer required	[62]
Cefotaxime vs. heparin	113, >65 yrs. (tunnelled cuffed catheters)	Yes (at 1 year, 68.7 vs. 31.3 %, *p* < 0.001)	Small sample, high baseline proportion infection	[63]
Cefotaxime vs. heparin	109, diabetic (tunnelled cuffed catheters)	Yes (at 1 year, 3.7 vs. 1.6/1000 catheter-days)	Small sample, majority of reduction attributable to Gram negative infections	[64]
46.7 % citrate vs. heparin^b^	210 (tunnelled cuffed catheters)	Yes (0.81 vs. 2.13/1000 catheter-days; *p* < 0.0001) Thrombosis reduced (RR 0.87, 95 %CI 0.83–0.93, *p* < 0.0001)	Thrombosis measured indirectly (alteplase use), no benefit in diabetics or in those with prevalent catheters	[65]
rtPA (1 of 3 sessions/week) vs. heparin (3 times/week)	225 (new HD lines)	Yes (0.40 vs. 1.37/1000 catheter-days; *p* = 0.02) Line failure reduced (20.0 vs. 34.8 %, *p* = 0.02)	RCT, patients and assessors blinded, high cost of rtPA	[66]
Taurolidine-citrate-heparin vs. heparin	565 (tunnelled cuffed catheters)	Yes (0.69 vs. 1.59/1000 catheter-days, *p* < 0.004)	Single centre, 2-year prospective observational study (not randomized)	[67]

*CFU* colony-forming unit, *mL* millilitre, *RCT* randomized controlled trial, *rtPA* recombinant tissue plasminogen activator

^a^All antibiotic lock studies compared drug and heparin to heparin alone

^b^Entire centre allocated to intervention for study period and compared to a control period. All studies prospective and randomized with 6-month follow-up unless otherwise indicated

Promising results have been reported with the use of trisodium citrate locks [65]. Citrate inhibits the formation of biofilms and is bactericidal to *Staphylococcal* species. High-concentration solutions have been reported effective in reducing infection, which may be related to inhibition of biofilm formation given its efficacy in preventing infection in patients with new catheters. In the prospective study by Winnett et al., there was an overall reduction in bacteremia when 46.7 % citrate locking solutions were used. However, subgroup analyses failed to show significance in infection reduction in diabetic patients or in those who had catheters present from before the intervention phase of the study. Another prospective open-label study of patients with pre-existing catheters failed to show a significant difference in CRBSI versus standard heparin therapy but was underpowered, and baseline rates of infection were very low during the study period [71]. As expected, most of the reductions were observed in Gram-positive species (MRSA, MSSA, CONS). With citrate solutions, there has been historical hesitance for its use due to the potential for cardiac arrhythmia owing to calcium sequestration by high concentrations of this agent. Following a case of cardiac arrest in the Netherlands [72], the FDA released a warning letter about high-concentration sodium citrate catheter locking in 2000, leading to an effective ban on use in the USA. Additionally, there is some in vitro data to suggest high-concentration citrate can cause protein precipitation and may be linked to pulmonary embolism [73]. However, this was never reported in trials testing citrate in its anti-infective properties [74].

More recently, several studies have examined taurolidine, a semi-synthetic amino acid, either alone or in combination with lower concentrations of citrate, as a locking solution. Taurolidine appears to exert antiseptic properties, with activity against both Gram-positive and negative species, as well as fungal pathogens in vitro [75]. A recent meta-analysis of three randomized prospective trials using taurolidine-citrate solutions showed significant reduction in CRBSI (RR 0.47, 95 %CI 0.25–0.89) [76]. However, unlike pure citrated solutions, infection reduction was attributable to decreased infections with Gram-negative species. No difference in exit site infection was observed. Another sequential prospective study using a combination taurolidine-citrate-heparin lock showed a reduction in *Staphylococcal* infections (see Table 7) [67].

Finally, significant reduction in bacteremia was noted in the PreCLOT trial, a Canadian randomized trial that compared recombinant tissue plasminogen activator (tPA) to heparin as a locking solution every one out of three HD sessions. The trial also reported a reduced risk of line failure with tPA, with no difference in adverse outcomes such as bleeding [66]. The cost of tPA is approximately 10-fold more than that of standard heparin, resulting in significant cost considerations for uptake. However, a recent cost effectiveness analysis showed that the extra cost of using tPA was partially offset by reduction in costs associated with catheter complications and represented a non-significant increase in yearly patient healthcare cost [77].

Together with other infection prevention strategies, locking solutions provide a possible means of reducing bacteremia further and warrant ongoing study (Table 8).

**Table 8:** A summary of preventative strategies showing significant reductions in CRBSI

Preventative strategy	Details of impact
Mupirocin nasal eradication of *S. aureus*	Reduction in *S. aureus* bacteremia
Exit site treatment (mupirocin/polysporin™)	Reduction in *S. aureus* and MRSA bacteremia and exit site infection
Alternate locking solutions	
46.7 % citrate [65]	Reduction in bacteremia attributable to *Staphylococcus* spp. Risk of cardiac arrhythmia
Taurolidine-citrate [76]	Reduction in risk of bacteremia attributable to Gram-negative spp.
Recombinant tPA [66]	Reduction in bacteremia while also significantly reducing catheter failure rate
Improved catheter hygiene technique [79] (Additional chlorhexidine and 70 % alcohol at catheter hub/exit at start and end of HD)	Sustained reduction in incident bacteremia and intravenous antibiotic starts during 1-year follow-up

## Conclusions

Patients with ESRD are prone to infection due to numerous individual and treatment-related factors including decreased immunity, dialysis-mediated immune dysfunction, repeated hospital exposure, repetitive venous access, and catheter biofilm formation. Although AVFs are associated with the lowest risks of infection, tunnelled cuffed catheters remain common in Canada, underscoring the importance of strategies to reduce adverse outcomes of CRBSI.

Several strategies have been proposed to reduce the risk and complications of infection in patients receiving HD via a catheter, with varying levels of success. The most successful strategies likely involve highly trained collaborative care teams that focus on adherence to specific care bundles, which have proven to be very effective in the prevention of recurrent bacterial infection. Collaborative care models that include early consultation of an infectious disease specialist increase guideline adherence for antimicrobial selection, ensure close monitoring of infected patients, and timely removal of vascular access, significantly reducing septic mortality. Standardizing this model may improve outcome in HD centres. Some catheter-locking solutions show promise for preventing bloodstream infections in patients with catheters, something not afforded with current heparin or low-dose citrate locks. The theoretical drawbacks of drug side effect and development of microbial resistance are potential caveats that require prospective study.

Other promising preventative strategies include nasal *S. aureus* eradication and the use of exit site antimicrobial agents. However, studies to date suggest these approaches may be limited by high rates of bacterial resistance and low sustained rates of bacterial eradication with short-term interventions. Given the high prevalence of catheter use for HD in Canada, further development and testing of new innovations for CRBSI prevention should be a healthcare research priority in Canada.

## Abbreviations

AVF: arteriovenous fistula
AVG: arteriovenous graft
CKD: chronic kidney disease
CONS: coagulase-negative *Staphylococcus*
CVC: central venous catheter
CRBSI: catheter-related bloodstream infection
eGFR: estimated glomerular filtration rate
ESRD: end-stage renal disease
HD: haemodialysis
IE: infective endocarditis
MRSA: methicillin-resistant *Staphylococcus aureus*
MSSA: methicillin-sensitive *Staphylococcus aureus*
tPa: tissue plasminogen activator
